# P-1304. Public Health Impact of Changes to Meningococcal Vaccination Platform in the United States

**DOI:** 10.1093/ofid/ofae631.1485

**Published:** 2025-01-29

**Authors:** Katharina Schley, Jessica Presa, Vincenza Snow, Kathleen Dooling, Alejandro D Cane, Paula Peyrani, Raymond Farkouh, Shannon M Sullivan, J Jaime Caro

**Affiliations:** Pfizer Pharma GmbH, Berlin, Berlin, Germany; Pfizer, Inc., Collegeville, Pennsylvania; Pfizer Vaccines, Collegeville, PA; Pfizer Inc., Collegeville, Pennsylvania; Pfizer, Collegeville, Pennsylvania; Pfizer, Inc, Collegeville, Pennsylvania; Pfizer, Inc., Collegeville, Pennsylvania; Evidera Inc., Ivry-sur-Seine Cedex, Ile-de-France, France; Evidera Inc., Waltham, Massachusetts

## Abstract

**Background:**

In the US, vaccination recommendations for the prevention of Invasive meningococcal disease (IMD) include routine MenACWY vaccination at 11-12 years (y) of age and a booster at 16 y and two doses of MenB vaccine as shared clinical decision making from 16-23 y. Recently a MenABCWY vaccine became available and was recommended when both MenACWY and MenB are to be given at the same visit. The aim of this study is to assess the public health impact of various alternative vaccine strategies compared to current ACIP vaccine recommendations.

**Methods:**

A static longitudinal cohort model was developed to estimate the impact of various vaccination strategies on IMD cases and deaths. A cohort of 11-y-olds was simulated for 50 y after vaccination. Vaccine uptake differed according to age and vaccination strategy.

**Results:**

The current strategy results in 364 cases (82 cases due to MenB and 282 cases due to MenCWY) in one cohort of 11-y-olds. Keeping the dose of MenACWY vaccine at 11 y and switching to two doses of MenABCWY vaccine at 16 and 17 y would additionally prevent 15 MenB and 6 MenCWY cases over 50 y compared to the current strategy. Removing the dose of MenACWY vaccine at 11 y and using two doses of MenABCWY vaccine at 16 and 17 y would prevent 22 additional MenB cases but lead to 73 additional MenCWY cases compared to the current strategy. In contrast, offering one dose of MenABCWY at 11 y and one dose at 16 would prevent 26 additional MenB cases and 4 additional MenCWY cases compared to the current strategy. Offering two doses of MenABCWY at 13 y (instead of 11) and 17 y would prevent 14 MenB cases while leading to 22 additional MenCWY cases.

**Conclusion:**

Our model suggests adding protection against serogroup B at 11 y would prevent additional cases of disease, while delaying adolescent protection against all serogroups of meningococcal disease will result in additional cases in the unprotected populations.

**Funding:** This study was funded by Pfizer Inc.

**Disclosures:**

**Katharina Schley, Dr. rer pol.**, Pfizer: Employee|Pfizer: Stocks/Bonds (Public Company) **Jessica Presa, MD**, Pfizer: Employee|Pfizer: Stocks/Bonds (Public Company) **Vincenza Snow, MD**, Pfizer: Employee|Pfizer: Stocks/Bonds (Public Company) **Kathleen Dooling, MD**, Pfizer: Employee|Pfizer: Stocks/Bonds (Public Company) **Alejandro D. Cane, MD, PhD**, Pfizer: Employee|Pfizer: Stocks/Bonds (Public Company)|Pfizer: Stocks/Bonds (Public Company) **Paula Peyrani, MD**, Pfizer: Employee|Pfizer: Stocks/Bonds (Public Company) **Raymond Farkouh, PhD**, Pfizer: Stocks/Bonds (Public Company) **Shannon M. Sullivan, PhD**, Evidera: Employee|Pfizer: Advisor/Consultant **J. Jaime Caro, MDCM, FRCPC, FACP**, Evidera: Employee|Pfizer: Advisor/Consultant

